# Aerosol emission from the respiratory tract: an analysis of aerosol generation from oxygen delivery systems

**DOI:** 10.1136/thoraxjnl-2021-217577

**Published:** 2021-11-04

**Authors:** Fergus W Hamilton, Florence K A Gregson, David T Arnold, Sadiyah Sheikh, Kirsty Ward, Jules Brown, Ed Moran, Carrie White, Anna J Morley, AERATOR Group, Bryan R Bzdek, Jonathan P Reid, Nicholas A Maskell, James William Dodd

**Affiliations:** 1 Infection Science, North Bristol NHS Trust, Westbury on Trym, UK; 2 MRC Integrative Epidemiology Unit, Bristol, UK; 3 Bristol Aerosol Research Centre, School of Chemistry, University of Bristol, Bristol, UK; 4 Academic Respiratory Unit, North Bristol NHS Trust, Westbury on Trym, UK; 5 Physiotherapy Department, North Bristol NHS Trust, Westbury on Trym, UK; 6 Anaesthetics and Intensive Care Department, North Bristol NHS Trust, Westbury on Trym, UK; 7 Infectious Diseases, North Bristol NHS Trust, Bristol, UK; 8 Research and Development, North Bristol NHS Trust, Westbury on Trym, UK

**Keywords:** non invasive ventilation, infection control, respiratory infection, viral infection

## Abstract

**Introduction:**

continuous positive airway pressure (CPAP) and high-flow nasal oxygen (HFNO) provide enhanced oxygen delivery and respiratory support for patients with severe COVID-19. CPAP and HFNO are currently designated as aerosol-generating procedures despite limited high-quality experimental data. We aimed to characterise aerosol emission from HFNO and CPAP and compare with breathing, speaking and coughing.

**Materials and methods:**

Healthy volunteers were recruited to breathe, speak and cough in ultra-clean, laminar flow theatres followed by using CPAP and HFNO. Aerosol emission was measured using two discrete methodologies, simultaneously. Hospitalised patients with COVID-19 had cough recorded using the same methodology on the infectious diseases ward.

**Results:**

In healthy volunteers (n=25 subjects; 531 measures), CPAP (with exhalation port filter) produced less aerosol than breathing, speaking and coughing (even with large >50 L/min face mask leaks). Coughing was associated with the highest aerosol emissions of any recorded activity. HFNO was associated with aerosol emission, however, this was from the machine. Generated particles were small (<1 µm), passing from the machine through the patient and to the detector without coalescence with respiratory aerosol, thereby unlikely to carry viral particles. More aerosol was generated in cough from patients with COVID-19 (n=8) than volunteers.

**Conclusions:**

In healthy volunteers, standard non-humidified CPAP is associated with less aerosol emission than breathing, speaking or coughing. Aerosol emission from the respiratory tract does not appear to be increased by HFNO. Although direct comparisons are complex, cough appears to be the main aerosol-generating risk out of all measured activities.

**Key messages**

**What is the key question?**

- Do high-flow nasal oxygen (HFNO) and CPAP produce clinically relevant aerosols?

**What is the bottom line?**

- In healthy volunteers, CPAP produced no aerosol, and HFNO produced no clinically relevant aerosol, while coughing was associated with significant aerosol production.

**Why read on?**

- Management of patients in respiratory failure with potentially infectious pathogens remains a complex area with little evidence. This paper provides some of the first high-quality data on potential risks associated with aerosol emissions.

## Introduction

The WHO describes disease transmission through three routes: physical contact, ‘droplet’ inhalation (larger particles which settle in a reasonably short distance) or ‘airborne’ (smaller particles which travel as aerosols on air currents, remaining in the air for longer and distributing over a wide area).^1^ SARS-CoV-2, the virus that causes COVID-19, can be transmitted via aerosols with aerosol emission being the putative mode of transmission for many super-spreading events.^2 3^ Although the exact size of aerosol particles responsible for airborne transmission (and the ability of virus to survive in these particles) continues to be debated, it is clear that the dispersion of particles smaller than 5 µm is largely determined by the room ventilation (air exchange) rate, thereby posing a potential risk to include those not in close contact, especially in poorly ventilated areas.^4^ Quantifying the concentration of particles of this size range is therefore critical for understanding the risk of disease transmission.

Traditionally, medical procedures are deemed as ‘aerosol generating’ when there is a perceived risk of increased generation of aerosol from the patients’ mucosa or respiratory tract compared with normal breathing, exposing staff and others in the vicinity to risk of inhalation of aerosolised airborne virus. For these aerosol-generating procedures (AGPs), an extra set of infection control precautions are mandated.^5–7^ These additional precautions often involve: segregating these patients from others, changing personal protective equipment to include FFP3 (or N95) masks that limit aerosol inhalation rather than fluid-resistant surgical masks (FRSMs), ensuring adequate ventilation, and allowing ‘fallow’ time between procedures to allow aerosol to disperse.

These mitigation strategies have significant impact on healthcare capacity, costs and potential harms; it is therefore critical to accurately identify whether these procedures truly do generate aerosol.^8^ Our aim was to identify whether procedures generate appreciable aerosol and whether the aerosol number concentration is lower than that generated by a cough. If so, the AGP is likely of low risk and misclassified.

Oxygen delivery and respiratory support including continuous positive airway pressure (CPAP) and high-flow nasal oxygen (HFNO) are used for the management of hypoxaemic respiratory failure complicating COVID-19 pneumonia. CPAP and HFNO are currently deemed AGPs by both the WHO and Public Health England (now the UK Health Security Agency), although the evidence for these recommendations is sparse.^5 7 9 10^ Current guidance stipulates the need to cohort these patients and universal FFP3 usage in any setting where a patient is receiving CPAP or HFNO. However, universal FFP3 usage is not currently recommended when caring for general inpatients with COVID-19, despite the potential risks from breathing, speaking and coughing in this setting.

In this study, we set out to quantify aerosol generation in both CPAP and HFNO and compare it with breathing, speaking and coughing without these supports.

## Methods

Full technical methods are in the online supplemental appendix S1. In brief, healthy volunteers were recruited in ultra-clean, laminar flow operating theatres and underwent a protocolised set of testing (breathing, speaking and coughing) under different oxygen delivery systems and without respiratory support. Participants were instructed to perform three voluntary coughs into the measuring funnel, moving their head away from the funnel after each cough.

10.1136/thoraxjnl-2021-217577.supp1 Supplementary data

Aerosol measurements were taken simultaneously by two separate devices, the Aerodynamic Particle Sizer (APS) and Optical Particle Sizer (OPS) (both manufactured by TSI), via a 3D-printed funnel and through 0.45 m of conductive silicone tubing. Both devices were included as they work on differing technologies and are able to detect aerosols of differing sizes (APS, 0.5–20 µm; OPS, 300 nm–10 µm).

Aerosol number concentrations were compared via Wilcoxon rank-sum tests on paired data, with a Bonferroni adjusted p value for multiple comparisons. Speaking and breathing were assessed as the average number concentration of aerosol during the activity, whereas the peak number concentration was recorded for coughing. Given the non-parametric nature of the data, we report median and IQR for all results.

COVID-19 Patients were recruited on the infectious disease ward and had measurements of cough taken using the same methodology. Each measurement was taken at the bedside in single occupancy negative pressure rooms that draw clean air from the ventilation system above. However, background aerosol concentration was significantly higher than in the operating theatres, precluding reliable measurements of breathing and speaking.

## Results

### Overall results and demographics

Thirty-three participants were recruited, of which 25 were healthy volunteers, and 8 were hospitalised patients with COVID-19. Thirteen (57%) of the volunteers were female, with a median age of 35 years (IQR 32–40 years), weight of 72 kg (IQR 64–79 kg), height of 1.74 m (IQR 1.64–1.79 m) and body mass index (BMI) of 23.6 kg/m^2^ (IQR 22.0–25.5 kg/m^2^).

Hospitalised patients with COVID-19 were older (mean age 55 years, IQR 49–59 years), with five men and three women. Height and weight were available for two patients: both were 170 cm tall; one weighed 85 kg (BMI: 29.4 kg), the other weighed 139 kg (BMI: 48.1 kg).

### Volunteer aerosol emission

Table 1 describes the number of times each activity was performed, and on how many volunteers, alongside aerosol emission for each activity. The number of activities does not match the number of participants, as some volunteers (n=6) repeated the assessments on a different day to check repeatability, and some measurements were only performed on certain participants.

**Table 1:** Aerosol emission produced across all activities in healthy volunteers

Oxygen delivery	Activity	Number of measurements	Aerosol emission (APS, particles/cm^3^)*	Aerosol emission (OPS, particles/cm^3^)*
Nil	Breathing	25	0.044 (0.022–0.08)	0.042 (0.023–0.125)
Nil	Speaking	25	0.088 (0.064–0.212)	0.121 (0.075–0.237)
Nil	Speaking with FRSM	23	0.03 (0.016–0.131)	0.038 (0.013–0.166)
Nil	Cough	25	1.52 (0.601–3.06)	2.14 (0.49–4.382)
Nil	Cough with FRSM	23	0.12 (0.06–0.555)	0.15 (0.06–0.57)
HFNO (60 L/min)	Breathing	20	1.861 (1.54–3.458)	2.921 (2.127–5.044)
HFNO (60 L/min)	Speaking	20	1.855 (1.201–2.359)	2.571 (1.65–3.255)
HFNO (60 L/min)	Cough	21	3.006 (2.597–5.525)	4.25 (3.011–6.41)
HFNO (60 L/min)	Cough with FRSM	10	0.63 (0.21–2.189)	0.75 (0.375–1.89)
CPAP at 15 mm Hg	Breathing sampling at area of greatest natural leak	20	0.013 (0.009–0.024)	0.012 (0.009–0.035)
CPAP at 15 mm Hg	Breathing sampling at exit port	20	0.002 (0–0.006)	0 (0–0.002)
CPAP at 15 mm Hg	Speaking sampling at exit port	8	0 (0–0.002)	0.001 (0–0.002)
CPAP at 15 mm Hg	Cough sampling at exit port	19	0.04 (0.01–0.06)	0.04 (0–0.105)
CPAP at 15 mm Hg	Cough sampling at leak	17	0.12 (0.06–0.72)	0.21 (0–0.99)
CPAP at 15 mm Hg	Removing CPAP mask	6	0.36 (0.195–0.57)	0.36 (0.27–0.6)

*This is the median IQR across individuals; average particles/cm^3^/s for continuous activities, peak particles/cm^3^ for sporadic activities.

APS, Aerodynamic Particle Sizer; FRSM, fluid-resistant surgical mask; HFNO, high-flow nasal oxygen; OPS, Optical Particle Sizer.

Correlation between the APS and OPS devices was high (r=0.98 unlogged, r=0.80 logged), despite the differing methodologies. Therefore, further analysis reports the APS figures in the text only, except where stated.

**Figure 1:**
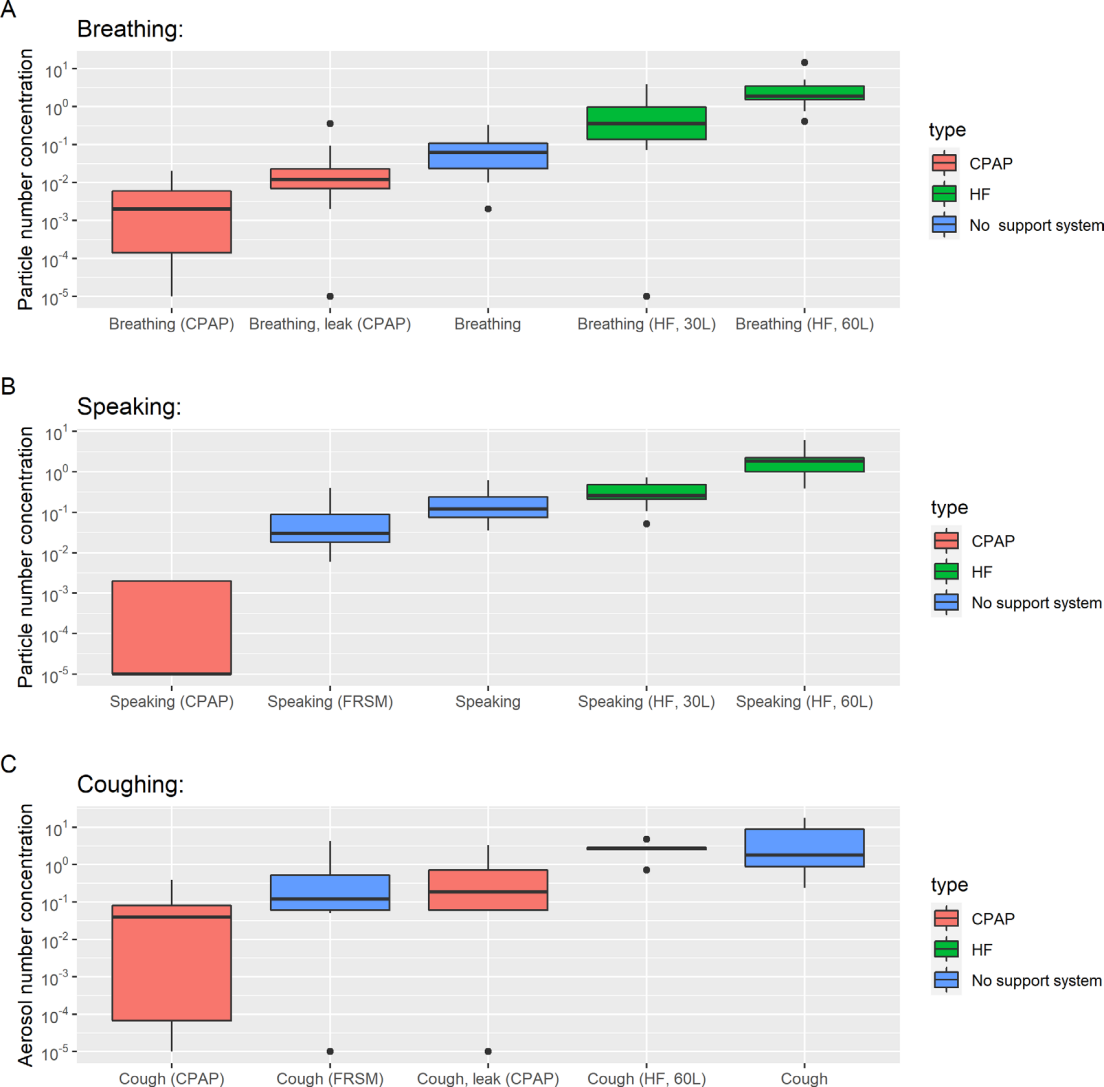
The aerosol number concentration sampled by an APS during baseline activities, CPAP or HFNO, reporting the mean concentration sampled during breathing (A) and speaking (B), and reporting the peak concentration sampled during coughs (C). Boxplots represent median and IQR. APS, Aerodynamic Particle Sizer; FRSM, fluid-resistant surgical mask; HFNO, high-flow nasal oxygen.

Figure 1A, B, C shows the aerosol number concentrations of each activity for volunteers, as reported by the APS (see online supplemental figure S4 for the OPS) and shows the clear variation in aerosol concentrations as well as the typical log-normal distribution. For baseline measurements, speaking produced more aerosol than breathing, and wearing an FRSM significantly reduced measured aerosol emission during both speaking (median 0.88 vs 0.03 particles/cm^3^, p<0.0001) and coughing (median 1.52 vs 0.12 particles/cm^3^, p<0.0001).

10.1136/thoraxjnl-2021-217577.supp2 Supplementary data

### Continuous positive airway pressure

As shown in figure 1, aerosol emission sampled from participants receiving CPAP is greatly reduced compared with baseline measurements while breathing, speaking and coughing. Even with a large induced face mask air leak (>50 L/min), the aerosol emission measured over that leak during coughing was lower than in participants not receiving CPAP (0.12 vs 1.52 particles/cm^3^; p<0.0001). At the filtered CPAP exit port, the aerosol emission was negligible and much reduced compared with those emitted during breathing, speaking or coughing in ambient room air (p<0.0001 for all comparisons).

Removal of the CPAP mask was associated with some aerosol emission, but this was significantly less than a cough in a healthy volunteer (peak of 0.36 particles/cm^3^ vs 1.52 particles/cm^3^, p<0.0001). In summary, CPAP was not associated with increased aerosolisation, but conversely was associated with much lower recorded aerosol number concentrations across all settings.

### High-flow nasal oxygen

Assessment of aerosol emission from HFNO was complex. Our initial experiment used a single HFNO machine, with details described below.

HFNO was associated with increased aerosol number concentrations compared with breathing ambient room air (median aerosol in HFNO 30 L/min, 0.277 particles/cm^3^; HFNO 60 L/min, 1.86 particles/cm^3^; ambient air 0.03 particles/cm^3^, p<0.0001 for all comparisons).

Higher flow rates (60 L/min) were associated with higher reported aerosol number concentrations than lower flow rates (30 L/min) for speaking (1.86 vs 0.246 particles/cm^3^, p<0.001), breathing (1.86 vs 0.277 particles/cm^3^, p<0.001), but not coughing (3.01 vs 2.96 particles/cm^3^, p=0.002), nor coughing with a surgical face mask (0.63 vs 0.24 particles/cm^3^, p=0.007), as both did not meet the Bonferroni corrected threshold (p=0.0004).

On review, the characteristics of the aerosol emissions during HFNO were not consistent with production of aerosol from the respiratory tract or mucosal surfaces, and aerosol was emitted even when the machine was unattached to the patient. We therefore performed a set of experiments to assess the source of this aerosol and their size distribution, with full experimental detail and results in the online supplemental appendix (see ‘Aerosol Concentrations Generated by HFNO’ and online supplemental figures S5–S8).

Importantly, we found that aerosol emission varied greatly among machines (two of four tested machines did not generate any aerosol), and that the size distribution of aerosol was not consistent with aerosol from the respiratory tract.

### Patients with COVID-19 versus healthy volunteers

In total, eight patients were recruited with COVID-19. Demographics of these patients are recorded in online supplemental table S2. Measurement of aerosol concentrations generated by these patients was technically difficult due to the acute clinical environment, infection control requirements and the" room's higher background aerosol number concentration, necessitating high efficiency particulate air (HEPA) filtration to reduce this concentration and allow respiratory aerosol measurements. Four participants were on standard, low-flow nasal oxygen, while the others were breathing room air. For four participants, the background aerosol concentration in the room was higher than median concentrations generated by speaking and breathing measured in the ultra-clean theatres, so we only report the aerosol emission from coughing.

**Figure 2:**
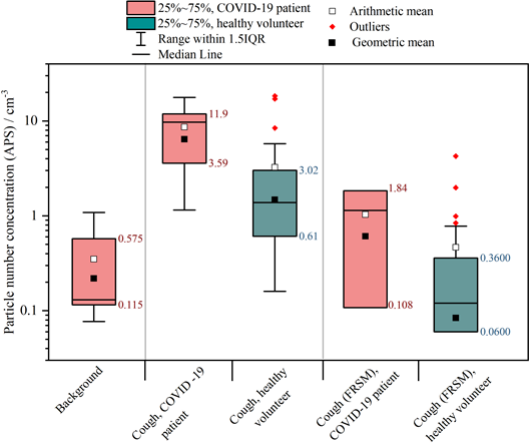
Box and whisker plot comparing the aerosol sampled by an APS when coughing in healthy subjects and by PCR-positive patients with COVID-19. APS, Aerodynamic Particle Sizer; FRSM, fluid-resistant surgical mask.

Figure 2 shows the aerosol emission recorded during coughing for both patients and volunteers. Compared with volunteers, patients with COVID-19 had higher aerosol emission when coughing (n=8; 10.5 vs 1.52 particles/cm^3^, p=0.002) and when coughing wearing an FRSM, although due to low numbers neither met Bonferroni correction (n=3, 0.94 vs 0.12 particles/cm^3^, p=0.03).

Importantly, the size distribution of aerosol particles in patients with COVID-19 was very similar to healthy volunteers (online supplemental figure S4). Breathing, speaking and coughing all generated aerosol particles in a log-normal size distribution with the peak in the 0.5–1 µm diameter range, consistent with previous reports of the size distribution of respiratory aerosol emissions.^11–14^ This supports the use of healthy volunteers as proxies for patients infected with SARS-CoV-2.

### Repeated measurements

For a subset of healthy volunteers (n=6), repeated measurements were made 1 month later. In total, there were 116 measurements repeated, (76 APS; 40 OPS). Correlation with the original measurement was moderate (r=0.71 on logged data), although this was driven by strong correlation in breathing (r=0.81), rather than speaking (r=0.17 on logged data) and coughing (r=0.38 on logged data) suggesting aerosol concentrations from breathing are relatively consistent for any individual recorded over a period of time, given the limitations inherent in the small numbers. In general, measurements on the second visit were marginally lower, which may represent slight differences in the experimental set-up.

Online supplemental figures S1 and S2 show these data, coloured by participant (S1) and activity (S2). Online supplemental figure S3 shows a Bland-Altman plot of this relationship.

**Figure 3:**
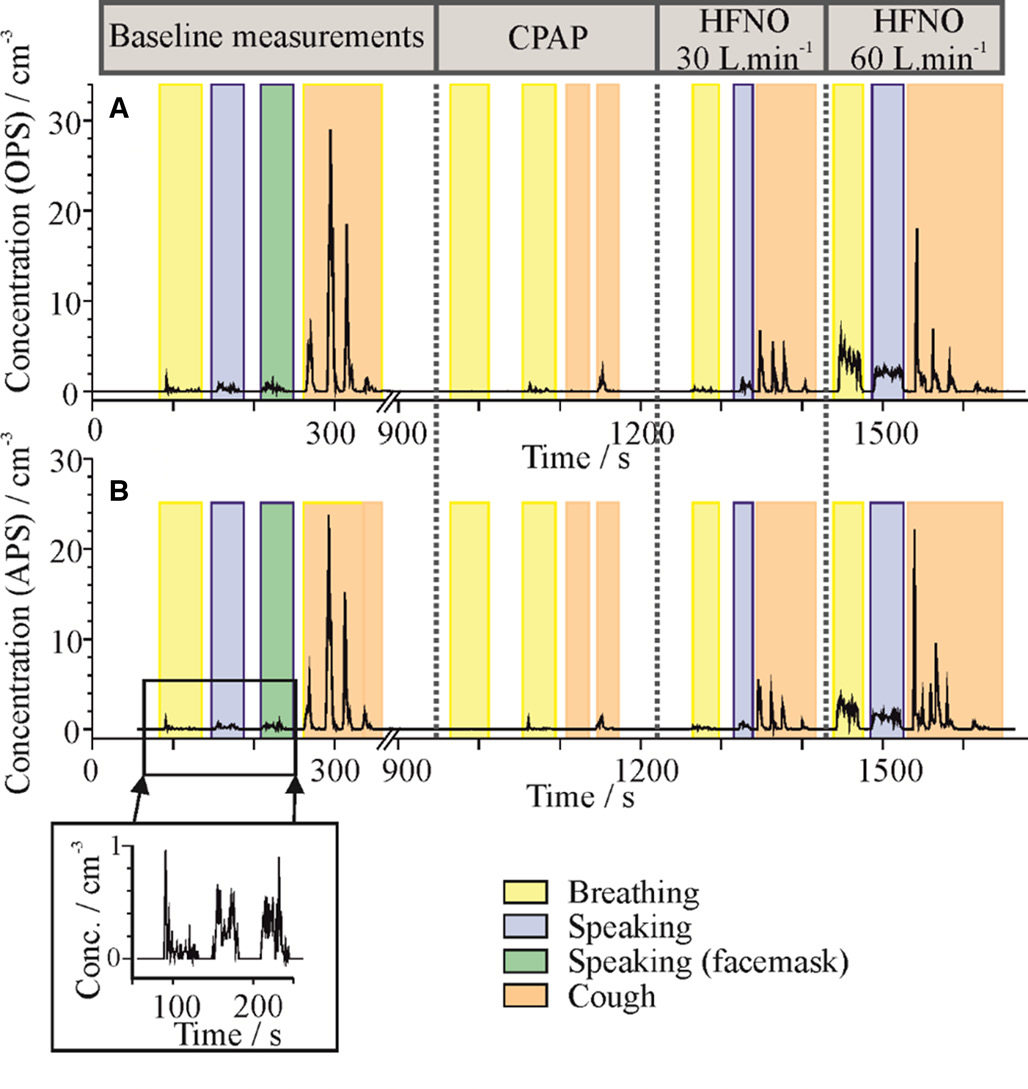
Example of the time series of OPS (A) and APS (B) number concentrations sampled during a measurement of one healthy subject performing baseline activities, followed by CPAP then HFNO. APS, Aerodynamic Particle Sizer; HFNO, high-flow nasal oxygen; OPS, Optical Particle Sizer.

## Discussion

### Summary

This study comprehensively characterised the aerosol generation during standard CPAP and HFNO procedures, as compared with normal breathing, speaking and coughing. CPAP delivered by face mask with exhalation filter was actually associated with lower aerosol number concentrations, even when large air leaks created around the CPAP face mask (>50 L/min) reflect the disruptions in CPAP of routine clinical care. For HFNO, aerosol concentrations were higher than baseline recordings. However, this additional aerosol was only present in some machines and largely disappeared with the use of a filter between the device and the patient. The size distribution of aerosol was unchanged when measured directly from the device or when attached to a patient, further supporting a non-biological origin.

Therefore, CPAP and HFNO should not be deemed AGPs, and provide no greater risk to healthcare staff relative to patients breathing, coughing and talking.

This is the first study to report on aerosol emission from patients with active COVID-19, with previous work on primates only.^15^ While the data suggest that peak aerosol concentrations from coughs are higher than those from healthy volunteers without COVID-19, the background aerosol concentration on the ward was too high to report data on speaking and breathing.

Our analysis shows that a single cough generates at least 10-fold more aerosol particles at the peak concentration relative to the mean concentration for speaking or breathing (median concentrations of 1.52 particles/cm^3^, 0.088 particles/cm^3^ and 0.03 particles/cm^3^ for cough, speaking and breathing, respectively, p<0.0001 for all comparisons).

In summary, our data (in concert with prior research on AGPs,^9 12^ epidemiological studies showing lower risk to staff working in intensive care^16–20^ and with viral loads higher earlier in infection, when patients are more often on the general wards^21^) suggest that risk of SARS-CoV-2 infection is not due to CPAP or HFNO generating infective aerosols. This has implications for infection and prevention control policy since aerosol generation appears greatest from patients with COVID-19 who are coughing.

### Strengths and weaknesses

This study has multiple strengths. First, it was performed in ultra-clean laminar flow theatres, with very low aerosol background concentrations, allowing accurate quantification and attribution of aerosol emission. Second, the strong correlation between both aerosol measurement modalities provides confidence that the aerosol measurements are reliable. Third, repeated measurements and the recruitment of patients with active COVID-19 allow greater interpretation of clinical implications. Finally, the protocol reflects usual clinical care and is directly translatable to the health service delivery.

However, there are some important weaknesses. First, measurement methodology employed by the APS and OPS uses relatively low flow rates (1 L/min and 5 L/min, respectively). These mean that very short, high-impact aerosol emissions (eg, cough) may be hard to quantify. However, this applies to all APS and OPS technology, and does not limit relative comparison between oxygen delivery systems. Second, our assessment of patients with COVID-19 is limited, as we only recruited hospitalised patients and were only able to reduce background aerosol emission enough to reliably measure cough. Although these data are limited, they suggest that aerosol emission from patients with COVID-19 is likely to be higher than in volunteers, and underlines the difficulties in making these measurements in real patients.

It is important to note that the majority of our measurements (like in all other studies so far) come from healthy volunteers. It is likely that demographics (weight, height, age) have some impact on aerosol emission, and therefore some caution must be taken in extrapolating the raw data on aerosol emission, although we have no reason to suspect the changes in aerosol emission seen with delivery systems would dramatically change.

Finally, our non-humidified CPAP system uses full face masks with exhalation filters, which are standard care in our hospital and in many NHS hospitals outside critical care, following national policy at the start of the pandemic.^22^ We cannot extrapolate to CPAP face masks without exhalation filters, although aerosol concentrations recorded from the face mask leak (unfiltered) were less than coughing without CPAP. As our system was unhumidified, we also cannot generalise to systems that use external humidification.

Similarly, the variability within the HFNO oxygen system used suggests that recorded aerosol emission may vary with device. As we only tested one manufacturer for each device, we cannot be sure that aerosol emission would differ with other manufacturers. However, as the mechanism of humidification and pressure generation is similar across different HFNO devices, it is likely that clinically relevant aerosol emission (eg, from the respiratory tract) is similar across devices. It is important to note that we cannot extrapolate to humidified CPAP devices, although we note the use of non-humidified CPAP is common in the management of patients with COVID-19 across many institutions.

We have chosen not to correct the reported particle concentrations sampled during each procedure to account for the effect of dilution by the airflow because the relative flow rate between each subject’s different exhalation events compared with CPAP and HFNO is ill-defined. Thus, the uncorrected aerosol number concentrations as sampled by the APS and OPS do not represent the absolute quantity of particles generated by each activity, but can be used as a measure of the risk to a healthcare worker in the vicinity of the activity.

As activities such as coughing are forceful and short lived, these were analysed separately to the continuous activities (eg, breathing): short, transient activities are observed as a rapid rise in the reported number concentration followed by a decay over a few sample measurements (typically equivalent to 10–15 s for a cough) as the aerosol dissipates from the sampling funnel and is diluted by the clean room air. While reporting the intensive property of concentration allows us to compare relative yields from AGPs, it is important to note that estimating absolute yields or fluxes (extensive properties) requires knowledge of the volumetric flow rates for the gas in which the aerosol is dispersed. These present an additional challenge to measure. Although it is possible to report the absolute number of particles counted by the instruments (given we know their sampling volumetric flow rates), we cannot conclude that this is equivalent to the total aerosol yield without knowing the volumetric flow rate at aerosol source (ie, participant’s mouth).

### Comparisons with previous literature

There are few published studies of aerosol generation from oxygen delivery systems and respiratory support. The most similar study was performed by Gaeckle *et al*, which measured protocolised respiratory support systems in volunteers.^9^ A similar protocol was used, although they also measured simple nasal cannulae and changes in respiration. Importantly, they reported a background aerosol concentration of ~0.060 particles/cm^3^ (compared with zero under laminar flow), higher than we report for many activities (including breathing and speaking with an FRSM). As well as a high background, the aerosol number concentration was highly variable in their study (see figure 4 and E3 from reference 11, and figure 3 here for comparison). This variability makes reporting accurate aerosol concentrations for short events (eg, a cough) challenging, as we noted in recruiting our patients with COVID-19.

Consistent with our study, Gaeckle *et al* reported non-invasive ventilation to be non-aerosol generating. However, by contrast, they did not identify increased aerosol emission with HFNO. A very recent study, performed by Wilson *et al,* 23 measured aerosol counts in 10 healthy volunteers in a chamber, attempting to collect all exhaled aerosol. Similar to our study, they found coughing produced large amounts of aerosol compared with breathing and speaking. However, in contrast to our study, they identified small increases in aerosol emission with both CPAP (2.6-fold with single circuit) and HFNO (2.3-fold) with normal breathing. However, during exertion, they identified a reduction in aerosol emission with both of these therapies compared with breathing unaided. As Wilson *et al* comment (on our preprint), the differing results likely reflect different approaches to measuring and recording peak and average aerosol emission, but the underlying results from both studies suggest that coughing represents a more significant source of aerosol than respiratory supports such as CPAP and HFNO.

The analysis presented in this paper, along with other work from our group identifying that intubation does not also generate significant aerosol,^12^ suggest that the current infection and prevention control aerosol risk stratification strategies based on procedures rather than time spent in contact with patients coughing with COVID-19 may be misplaced.

### Implications for clinical practice and policy

This study strongly supports re-evaluation of guidance removing CPAP as a high-risk AGP, with implications for more efficient delivery of NHS services. However, given that patients who receive acute respiratory support for COVID-19 are often acutely unwell and cough, the risk of aerosolisation of SARS-CoV-2 may be significant, complicating the policy changes. This work supports a re-evaluation of focusing solely on AGPs as potential risky events, and a shift towards focusing on the patient.

## Conclusions

Non-humidified CPAP delivered via a filtered mask actually reduces aerosol emission compared with normal breathing. HFNO does generate additional aerosol, however this aerosol is generated from the machine and not the patient, and is unlikely to pose extra clinical risk given the size (<1 µm). Cough appears to generate significant aerosols in a size range compatible with airborne transmission of SARS-CoV-2. Policy around aerosol generation and infection control should be updated to reflect these adjusted risks.

## Data Availability

All data relevant to the study are included in the article or uploaded as supplemental information. Anonymised aerosol data from the AERATOR Study will be submitted to the Bristol data repository (data.bris.ac.uk) on completion of the full study.
