# Supplementary material for: Aerosol emission from the respiratory tract: an analysis of aerosol generation from oxygen delivery systems
Source: Thorax. 2021 Nov 4;77(3):276–82. doi: 10.1136/thoraxjnl-2021-217577 (PMC8867281; doi:10.1136/thoraxjnl-2021-217577)
Supplement: Supplementary data [file thoraxjnl-2021-217577supp001.pdf]

Aerosol emission from the respiratory tract: an analysis of aerosol generation from oxygen delivery systems.

SUPPLEMENTARY APPENDIX 1

Hamilton F*[1,2], Gregson F*[3], Arnold D[4], Sheikh S[3], Ward K[5], Brown J[6], Moran E[1],White C[7] AERATOR group, Bzdek BR[3], Reid JP[3], Maskell N[4#], Dodd JW[4#]

*These authors contributed equally

#These authors contributed equally

1. Infection Sciences, North Bristol NHS Trust, BS10 5NB
2. MRC Integrative Epidemiology Unit, University of Bristol, BS8 2HW
3. Bristol Aerosol Research Centre, School of Chemistry, University of Bristol, BS8 1TS
4. Academic Respiratory Unit, University of Bristol, BS10 5NB
5. Physiotherapy Department, North Bristol NHS Trust, BS10 5NB
6. Intensive Care Unit, North Bristol NHS Trust, BS10 5NB
7. Research and Innovation, North Bristol NHS Trust, BS10 5NB

AERATOR group (in alphabetical order): The AERATOR group consists of (in alphabetical order): Arnold, D; Brown, J; Bzdek, B; Davidson, A; Dodd, JW; Gormley M; Gregson, F; Hamilton, F; Maskell, N; Murray, J; Keller, J; Pickering, A.E; Reid, J; Sheikh, S; Shrimpton, A.

Corresponding author:

James Dodd (james.dodd@bristol.ac.uk)

Full Technical Methods

Study design

This was performed as part of the wider AERATOR study to assess the risk of aerosolised transmission of SARS-CoV-2 in healthcare settings. Ethical approval was given by the North West Research Ethics Committee (Ref: 20/NW/0393, HRA Approved 18/9/20).

Aerosol measurement

Aerosol measurements were recorded using two devices simultaneously: an Optical Particle Sizer (OPS) and Aerodynamic Particle Sizer (APS). Technical specifications were detailed in a previous publication from our group but are replicated here.[1] The key difference was the use of a shorter length of sampling tube (0.45m) from the sampling funnel through to the instrument inlet as patients were seated for this experiment, not supine.

The APS (TSI Incorporated, model 3321, Shoreview, NM, USA) measures aerosol at a sampling flow rate of 1 L min^{-1} with accompanying sheath flow of 4 L min^{-1}. The APS reports the aerodynamic size of particles in an aerosol plume, size-resolving aerosol number concentration into 52 size bins ranging from 0.5 µm to 20 µm in diameter with a time integration of 1 s. The size bins are equally spaced in log(diameter) space, apart from the smallest size bin (0.5 - 0.523 µm).

The OPS (TSI Incorporated, model 3330, Shoreview, NM, USA) samples air at 1 L min^{-1} and detects particles by laser optical scattering. The OPS reports the particle number concentration and optical size distribution within the diameter range 300 nm to 10 µm with a time resolution of 1 second. The OPS is widely used for aerosol studies from laboratories / clean rooms to more demanding outdoor environments. It is calibrated by the manufacturer using polystyrene latex spheres and its performance conforms to the ISO standard 21501-4:2018. The reported optical size of the particles is based on an assumed refractive index of pure water at 600 nm wavelength (1.333).

Both the APS and OPS were connected to the same sampling funnel, which was 3D printed (RAISE3D Pro2 Printer, 3DGBIRE, Chorley, UK) from PLA with a maximum diameter of 150 mm, cone height of 90 mm with a 10-mm exit port. Two conductive silicone sampling tubes of 0.3 m length and internal diameter 4.8 mm (3001788, TSI) were connected to the neck of the sampling funnel, with one connected to the APS and the other to the OPS.

For baseline measurements and HFNO, the funnel was placed such that the top of the funnel cone was 1 cm from the participant's forehead, and the sampling apex of the funnel was 10 cm from the

participant's mouth. For CPAP the sampling apex of the funnel was 10 cm from the exit port or area of greatest facemask leak. Supplement 1 includes some images from the set up to aid visualisation.

Environmental set up and patient recruitment

Observations were performed in two settings: healthy volunteers were recruited in an ultra-clean laminar flow operating theatre (EXFLOW 32, Howarth Air Technology, Farnworth, UK) with high efficiency particulate air (HEPA) filtration and an air supply rate of 1200 m^3/s. This ventilation system has a canopy 'clean zone' where surgical procedures are performed; the air circulation velocity is 0.2 $m.s^{-1}$ at 1 m above the floor below the canopy and produces 500–650 air changes per hour. All aerosol recordings were performed under the canopy, and the background aerosol concentration was sampled prior to each measurement for a mean sampling duration of 43 s, with mean background number concentrations of 0.00187 (SD 0.00271) cm^{-3} and 0.00330 (SD 0.00395) cm^{-3} reported by the APS and OPS, respectively. Air temperature in theatres was set to 20 °C and humidity between 40 and 60%.

The NIV machine used for delivering CPAP was the Phillips Trilogy 202 and the HFNO machine was the Fisher and Paykel Airvo 2. They were used according to manufacturer instructions. The CPAP circuits used were Armstrong Medical: AMVC1792/032 and Respironics: 1065830, CPAP full face masks were ResMed: AcuCare F1-0 and X, and the nasal cannula used were Fisher and Paykel Optiflow OPT944. The CPAP masks were unvented, as is standard UK practice, with a filtered exhalation valve. At the start of the pandemic, NHS England issued guidance recommending against external humidification, so our CPAP set up was unhumidified.[2]

Patients hospitalised with PCR-positive COVID-19 pneumonia were recruited and measurements taken in negative pressure ventilated side-rooms in the infectious disease ward. To reduce the background aerosol number concentration sufficient to allow measurements of baseline procedures in the ward setting, we used a portable HEPA filter (PUREAir R150, PUREAir Limited) with an airflow rate of 1500 m^3/hour for some patients. This reduced the background concentration for most measurements, although the average background number concentration was 0.351 (SD 0.382) cm^{-3} and 0.407 (SD 0.472) cm^{-3} for the APS and OPS, respectively, still significantly higher than in the laminar flow theatres. Where available, participant height and weight were recorded.

Healthy volunteers were recruited and invited to undergo a protocolised sequence of procedures in laminar flow environment of an operating theatre (see below for full procedural list). These included: tidal breathing, speaking (with/without mask), coughing (with/without mask), and

receiving CPAP (non humidified, full face mask, Continuous Positive Airway Pressure 15cmH_2O), and HFNO (High flow nasal oxygen). All procedures were performed in the seated position. For some participants, additional measurements were taken (e.g. speaking during HFNO, taking off the CPAP mask). Height and weight were recorded for all volunteers. A sample of the volunteers were invited to have a second measurement on a different date to ensure replicability. For CPAP, sampling was at the filtered exit port of the facemask, and at the point of maximum leak from the mask (measured by an experienced operator). If the maximum leak was less than 50 L/min, a leak was generated by the operator and measurement performed as close as possible to this leak. As with baseline measurements, speaking, breathing, and coughing were recorded during CPAP. Finally, measurements were made as the mask was removed for a small number of participants. CPAP settings were set to our hospital standard (15cm H_2O pressure), after initial scoping measurements and earlier research found no difference in aerosol emission with changing pressure, oxygen and humidity settings.

For HFNO, As with CPAP, measurements were made during tidal breathing, speaking, and coughing. For some participants, we tested the effect of wearing a surgical facemask over the HFNO nasal cannula on aerosol emission. As described in detail in supplement (Supplement S2), we also performed a set of measurements and experiments with four separate HFNO machines in order to identify the source of aerosol that we recorded during our study.

Hospitalised patients with COVID-19 were recruited by study members and had simple baseline measurements performed (e.g. speaking, breathing, and coughing, both with and without surgical facemasks). However, as background aerosol concentration was too high to reliably report aerosol emission from breathing and speaking, we only report aerosol emission from coughing.

Statistical analysis

Aerosol generation differs greatly among people, with an approximate log-normal distribution in number concentration.[3,4] As such, our analysis focussed on comparing the relative aerosol number concentrations from different procedures performed by each individual. We report the number concentration, an intensive property that does not depend on scale (i.e. is independent of the time or volume sampled) as reported by the instruments measured over a sample period, selected to be 1 s. We have reported one of two parameters for each activity: either the peak particle number concentration reported across the full number of samples of the measurement for single, forced exhalations such as coughing (cm^{-3}); or, the mean particle number concentration reported as the

average across all samples for continuous activities such as breathing or speaking (cm^{-3}). We then visualised size distributions of aerosol emission across the volunteers and compared aerosol emission across activities. As the data are non-parametric, we present median and interquartile ranges for results, and comparisons were made using the Wilcox-Sum rank test. Given the potential number of comparisons, we Bonferroni adjusted the p-value (0.05/120, p = 0.0004) to reduce false positive associations

Data analysis was performed by collating raw data of sampled aerosol concentration output by the APS and OPS instruments using Aerosol Instrument Manager 9.0 (TSI Incorporated, Shoreview, NM, USA) and Microsoft Excel. A custom-written software in LabVIEW (National Instruments, Texas, USA) was used to automate the analysis process for increased efficiency. For the PCR-positive hospitalised patients with COVID-19, the mean background aerosol number concentration was subtracted from the sampled aerosol number concentration for each activity to account for the non-zero background and to allow comparison with the data from healthy subjects collected under laminar flow. Formal statistical analysis was performed using R 4.0.3 (R foundation for Statistical Computing, Vienna). As a secondary objective, we wanted to test whether aerosol emission was different for patients with COVID-19 rather than healthy volunteers. For this, we compared activities in hospitalised patients with COVID-19 and in healthy controls. Finally, we tested a subset of volunteers twice, on different days and with different operators, to assess the intra-person variability in aerosol emission.

List of procedure, plan and set up:

Aerosol instruments on (baseline 30 s)	Measurement position
Breathe into funnel (30 s)	Funnel 10cm from mouth
STOP step back from funnel	
Speak into funnel (30 s) ~ 75 dB	Funnel 10cm from mouth
STOP step back from funnel	
Speak (30 s) **WITH FRSM** ~ 75 dB	Funnel 10cm from mouth

STOP step back from funnel	
Cough, then step back for 20 seconds	Funnel 10cm from mouth
Cough, then step back for 20 seconds	Funnel 10cm from mouth
Cough, then step back for 20 seconds	Funnel 10cm from mouth
Cough, **WITH FRSM**, then step back for 20 seconds	Funnel 10cm from mouth
STOP step back from funnel + place CPAP mask on	
Manouvere so CPAP exit port in funnel (30s) + breathing	CPAP exhalation port in funnel
STOP step back from funnel	
Manouvere so CPAP area of greatest leak in funnel (30s) + breathing	Funnel 10cm from area of greatest leak
STOP step back from funnel	
Manouvere so CPAP exit port in funnel (30s) + speaking	CPAP exhalation port in funnel
STOP step back from funnel	
Manouvere so CPAP area of greatest leak in funnel (30s) + speaking	Funnel 10cm from area of greatest leak
STOP step back from funnel	
Manouver so CPAP exit port in funnel, cough, then stand back 20 seconds	CPAP exhalation port in funnel
STOP step back from funnel	
Manouver so CPAP exit port in funnel, cough, then stand back 20 seconds	CPAP exhalation port in funnel
STOP step back from funnel	

Manouvere so CPAP area of greatest leak in funnel, cough, then stand back 20 seconds (30s)	Funnel 10cm from area of greatest leak
STOP step back from funnel	
Manouver so CPAP area of greatest leak in funnel, cough, then stand back 20 seconds	Funnel 10cm from area of greatest leak
STOP step back from funnel - remove CPAP mask	
Place NHF02 on at low flow with entrained air - measure tidal breathing (30s)	Funnel 10cm from mouth
STOP step back from funnel	
Place NHF02 on at low flow (20L) with entrained air - measure tidal breathing (30s)	Funnel 10cm from mouth
STOP step back from funnel	
Place NHF02 on at high flow (60L) with entrained air - measure tidal breathing (30s)	Funnel 10cm from mouth
STOP step back from funnel	
Place NHF02 on at high flow (60L) with entrained air - speaking (30s)	Funnel 10cm from mouth
STOP step back from funnel	
Place NHF02 on at high flow (60L) with entrained air - speaking (30s) + FRSM	Funnel 10cm from mouth
STOP step back from funnel	
Cough, then step back for 20 seconds (on 60L/min)	Funnel 10cm from mouth
Cough, then step back for 20 seconds (on 60L/min)	Funnel 10cm from mouth
Cough, then step back for 20 seconds (on 60L/min)	Funnel 10cm from mouth
STOP step back from funnel	

Note 1: Because coughs tend to decrease in strength, for some participants, we did a cough wearing a FRSM first in some participants.

Note 2: Speaking was asking patients to count from 1-100, at a set cadence and aiming for a similar volume between participants.

<u>Images of the set up:</u>

Figure S1: Line drawing of the set up in theatres from above. **A**: is the volunteer, **B** is the measuring equipment table, and **C** represents the limits of the laminar flow canopy.

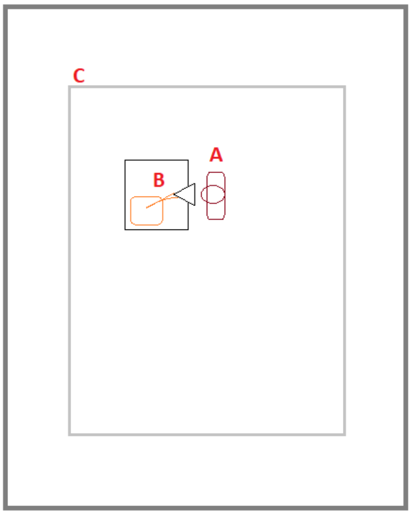

Figure S2: Image of the measuring equipment table, including **A: Funnel,** connected to **B: Silicon Tubing**, which connects to **C: Optical Particle Sizer** and **D: Aerodynamic Particle Sizer.**

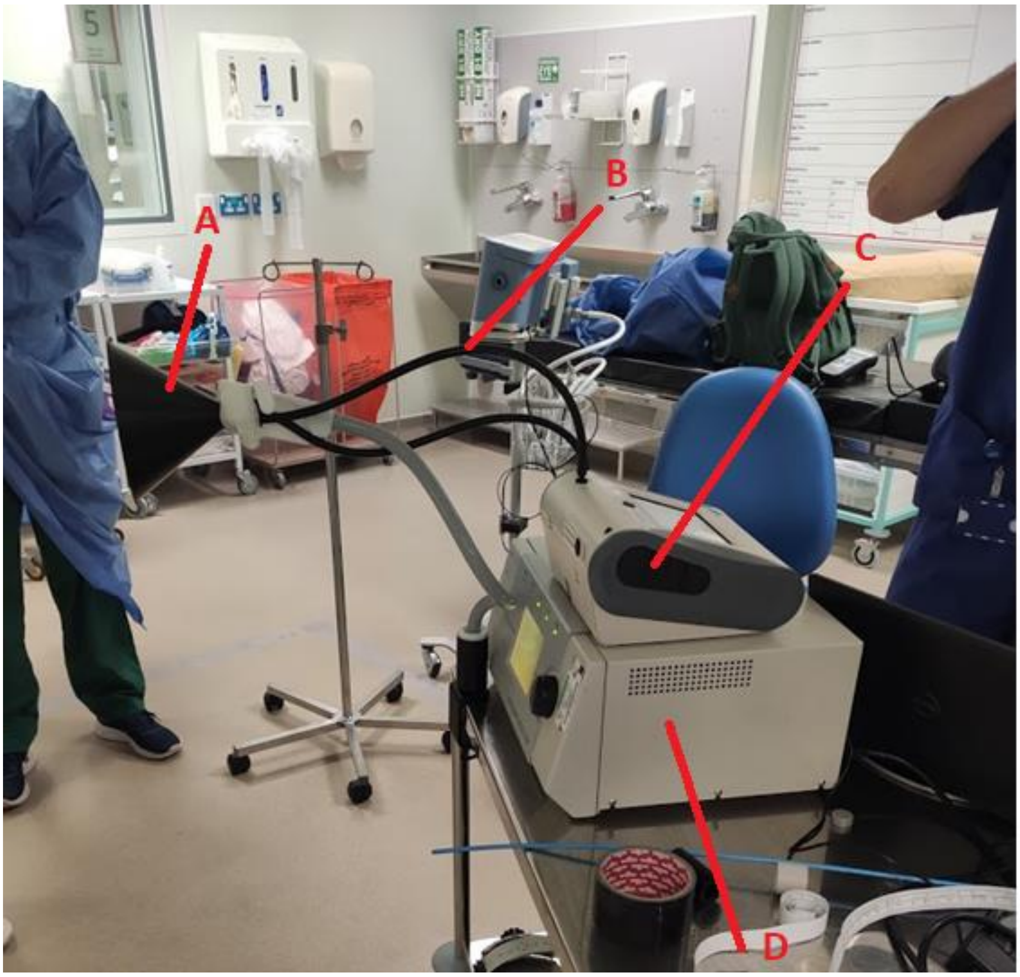

Figure S3: An example of someone receiving CPAP with the exit port facing the input funnel.

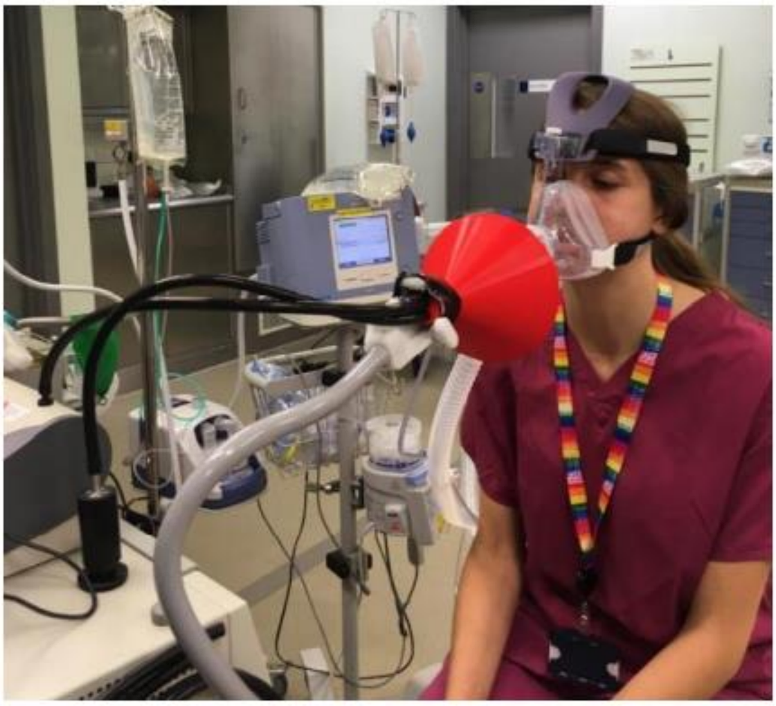

1. Brown J, Gregson FKA, Shrimpton A, et al. A quantitative evaluation of aerosol generation during tracheal intubation and extubation. Anaesthesia 2021;76(2):174–81.
2. England NHS. Guidance for the role and use of non-invasive respiratory support in adult patients with coronavirus (confirmed or suspected). Available from: https://amhp.org.uk/app/uploads/2020/03/Guidance-Respiratory-Support.pdf
3. Gregson, Watson, Orton, et al. Comparing the Respirable Aerosol Concentrations and Particle Size Distributions Generated by Singing, Speaking and Breathing. ChemRxiv [Internet] 2020 [cited 2021 Jun 24];Available from: https://chemrxiv.org/articles/preprint/Comparing_the_Respirable_Aerosol_Concentrations_and_Particle_Size_Distributions_Generated_by_Singing_Speaking_and_Breathing/12789221
4. Asadi S, Wexler AS, Cappa CD, Barreda S, Bouvier NM, Ristenpart WD. Aerosol emission and superemission during human speech increase with voice loudness. Sci Rep 2019;9(1):2348.
